# Persistent Hyperdopaminergia Decreases the Peak Frequency of Hippocampal Theta Oscillations during Quiet Waking and REM Sleep

**DOI:** 10.1371/journal.pone.0005238

**Published:** 2009-04-21

**Authors:** Kafui Dzirasa, Lucas M. Santos, Sidarta Ribeiro, Jennifer Stapleton, Raul R. Gainetdinov, Marc G. Caron, Miguel A. L. Nicolelis

**Affiliations:** 1 Department of Neurobiology, Duke University Medical Center, Durham, North Carolina, United States of America; 2 Department of Biomedical Engineering, Duke University Medical Center, Durham, North Carolina, United States of America; 3 Department of Psychological and Brain Sciences, Duke University Medical Center, Durham, North Carolina, United States of America; 4 Center for Neuroengineering, Duke University Medical Center, Durham, North Carolina, United States of America; 5 Department of Cell Biology, Duke University Medical Center, Durham, North Carolina, United States of America; 6 Department of Neuroscience and Brain Technologies, Italian Institute of Technology, Genova, Italy; 7 Edmond and Lily Safra International Institute of Neuroscience of Natal Edmond and Lily Safra (ELS-IINN), Natal, Brazil; Freie Universitaet Berlin, Germany

## Abstract

Long-term changes in dopaminergic signaling are thought to underlie the pathophysiology of a number of psychiatric disorders. Several conditions are associated with cognitive deficits such as disturbances in attention processes and learning and memory, suggesting that persistent changes in dopaminergic signaling may alter neural mechanisms underlying these processes. Dopamine transporter knockout (DAT-KO) mice exhibit a persistent five-fold increase in extracellular dopamine levels. Here, we demonstrate that DAT-KO mice display lower hippocampal theta oscillation frequencies during baseline periods of waking and rapid-eye movement sleep. These altered theta oscillations are not reversed via treatment with the antidopaminergic agent haloperidol. Thus, we propose that persistent hyperdopaminergia, together with secondary alterations in other neuromodulatory systems, results in lower frequency activity in neural systems responsible for various cognitive processes.

## Introduction

Hippocampal theta oscillations (HTO) are prominent local field potential oscillations generated by the brain, and occur within the 4–9 Hz frequency range [1], [2]. These oscillations are especially prominent during periods of exploration and rapid-eye-movement (REM) sleep [1]–[3], and play a critical role in high-end cognitive processes such as spatial learning, fear conditioning, and attention [4]–[6]. Many studies have been aimed at elucidating the neuromodulatory systems responsible for the generation and modulation of HTO's; however, the central methodologies employed across these studies have classically focused on using pharmacologic agents to acutely manipulate signaling within neuromodulatory systems [7]–[10]. Given the growing body of evidence suggesting that persistent changes in neuromodulatory systems underlie the behavioral and cognitive deficits observed across several neuropsychiatric disorders [11]–[15], there is increased demand for understanding how persistent changes in neuromodulatory systems alter these brain oscillations [16].

The neurotransmitter dopamine (DA) is critically involved in regulating neural processes responsible for complex movements, emotions, attention, and arousal and sleep states [17]–[19]. Acute administration of psychostimulants or direct DA receptor agonists generate high frequency HTO's in rats [20]. Importantly, these agents modulate extracellular dopamine levels [21]. Despite the clear effects of acute hyperdopaminergia on HTO's, influences of persistent hyperdopaminergia on HTO's are obscure. This is an important omission given that long-term changes in DA signaling are thought to underlie, at least in part, the pathophysiology of attention-deficit-hyperactivity disorder (ADHD) [11], schizophrenia [12] and bipolar disorder [13].

DA transporter (DAT) knockout (KO) mice lack the gene encoding the plasma membrane transporter that regulates spatial and temporal DA signaling at the synapse. Due to loss of the DAT, these mutants exhibit a persistent 5-fold increase in extracellular DA levels [21], and show locomotor hyperactivity, deficits in sensorimotor gating, and impaired learning and memory [22]–[25]. Here we demonstrate that DAT-KO mice display significantly lower HTO frequencies during baseline waking and REM sleep periods. Additionally, we show that the altered HTO's observed in DAT-KO mice are not corrected via treatment with haloperidol. Thus, we propose that persistent hyperdopaminergia and its associated secondary changes in other neuromodulatory systems ultimately results in lower frequency activity in neural systems responsible for high-end cognitive processes.

## Materials and Methods

### Animals

The WT and DAT-KO littermates were generated from heterozygotes that had been backcrossed over twenty generations onto the C57BL/6J background. Mice were housed three-five/cage and maintained in a humidity- and temperature-controlled room with standard lab chow and water available *ad libitum*. Thirteen male WT mice and eleven DAT-KO mice were separated into individual cages, and surgically implanted with electrodes and electromyographic (EMG) wires. Recording experiments were conducted following a one week recovery. All studies were conducted with approved protocols from the Duke University Institutional Animal Care and Use Committee and were in accordance with the NIH guidelines for the Care and Use of Laboratory Animals.

### Surgery

Adult mice (20–35 weeks) were anesthetized with a Ketamine (100 mg/kg)/Xylazine (10 mg/kg) solution, placed in a stereotaxic device, and ground screws were secured to the cranium. Tungsten microwire array electrodes were implanted through a small cranial window into the dorsal hippocampus (stereotaxic coordinates: −2.3 mm anterior posterior, 1.6 mm mediolateral and 1.8 mm dorsoventral from bregma), and anchored to ground screws using dental acrylic. Tungsten EMG wires were placed into the trapezius muscle, and the skin was closed using surgical sutures.

### Experimental set-up for electrophysiological recordings

All experiments were conducted in a recording chamber, consisting of an empty cage bottom (11.5 in×7 in×4.5 in) marked into six equal sections. Gross locomotor activity was determined by the number of section crosses normalized to total wake time during the recording period.

### Data acquisition

Local field potentials (LFPs) were preamplified (500×), filtered (0.3–400 Hz), and digitized at 500 Hz using a Digital Acquisition card (National Instruments, Austin, TX) and a Multi-Neuron Acquisition Processor (Plexon, Dallas, TX). Behaviors were recorded with a video cassette recorder. Video images were loaded onto a cassette recorder and synchronized with the neural recordings using a millisecond-precision timer.

### Behavioral state and total theta power identification

Behavioral states were identified by combining two-dimensional state map and EMG cluster analysis as previously described [19]. Briefly, the state map generated cluster separation based on the high amplitude theta (4–9 Hz) and gamma (33–55 Hz) oscillations characteristic of REM sleep, the absence of gamma oscillations and the high amplitude delta (1–4 Hz) characteristic of SWS, and the high amplitude gamma oscillations and theta oscillations characteristic of waking. Minor scoring errors typically occurred in differentiating REM sleep from active exploration, thus EMG analysis was used to identify periods of atonia consistent with REM sleep, and combined with the two-dimensional state map cluster scoring method for all sleep experiments conducted in this study. LFP's were simultaneously recorded from 8 implanted electrodes, and the LFP which produced the best cluster separation was used for all experiments presented here. Importantly, our method did not control for the layer of hippocampus in which the recording electrode used for analysis was located. This is an important confound because the amplitude of theta oscillations change as a function of depth in hippocampus [2]. For instance, our experiments in which five WT mice were recorded in the novel environment, habituated environment, and during REM sleep reveal a high coefficient of variance (CV) in the maximum theta power measured across animals (CV = 0.49, 0.52, and 0.52, for recordings in the novel environment, habituated environment, and during REM sleep, respectively). Additionally, our experiments in five DAT-KO mice reveal a similarly high coefficient of variation (CV) in the maximum theta power measured across animals (CV = 0.56, 0.49, and 0.55, for recordings in the novel environment, habituated environment, and during REM sleep, respectively). However, we found that by normalizing the maximum mean theta power measured during the waking periods by that recorded during REM sleep for each animals, we were able to reduce the CV measured across animals by up to 80% (CV = 0.0*9/0.15*, and 0.0*9/0.11* for theta power measured in the *novel cage/home cage* in WT and DAT-KO mice, respectively). Importantly, the mean power spectrum observed during REM sleep remained unchanged when DAT-KO and WT mice were treated with drugs, as well as during 12-hour experimental recordings conducted in their home cage [19]. These results indicate that the peak theta power observed during REM sleep can be utilized effectively as the baseline variable to correct for changes in theta wave amplitude recorded from different depths of hippocampus.

### Determination of peak theta frequency

First, all data segments with amplitude saturation were discarded from the dataset (2% of the total data per mouse). Using Matlab (MathWorks, Natick, MA), a sliding window Fourier transform was applied to the LFP signal using a 2 sec window with a 1 sec step. The Fourier transform parameters were chosen to allow for a frequency resolution of 0.5 Hz. Subsequently, LFP power spectra were then averaged across each period of interest (at least 2 total minutes for REM sleep, 10 total minutes for baseline waking, and 10 total minutes for active exploration), and the frequency at which the maximum spectral power occurred in the theta frequency (4–9 Hz) range was identified. For experiments examining REM sleep, we also determined the peak theta frequency using Fourier transform parameters chosen to allow for a frequency resolution of 0.25 Hz. The instantaneous phase values of theta oscillations shift throughout the depth of hippocampus; however, oscillations are phase locked across layers [2]. Thus, unlike measurements of theta power, the peak theta frequency is independent of which layer the recording electrode is located.

### Drug treatments during electrophysiological measurements

A 0.3 mg/kg (i.p.) dose of haloperidol (HAL) was chosen because it attenuates behavioral hyperactivity in novelty-exposed DAT-KO animals to a degree similar to that observed in untreated habituated WT mice [19]. d-Amphetamine (AMPH) was given at 3.0 mg/kg (i.p.) because it increased extracellular DA levels and induced behavioral hyperactivity in WT mice to similar degrees as those observed in untreated DAT-KO mice [26]. The serotonin selective reuptake inhibitor Fluoxetine (FLU) was given at 20 mg/kg (i.p.).

### Statistics

The data are presented as means and standard errors of the mean. The electrophysiological data were analyzed by two-way ANOVA, followed by Newman-Keuls (α = 0.05) tests for comparisons within genotype, and Student t-test for comparisons between genotypes. The behavioral data were analyzed by two-way ANOVA, followed by Student t-test for single comparisons. In all cases *p*<0.05 was considered significant.

## Results

### Hyperdopaminergic mice show alterations in baseline hippocampal theta oscillations

**Figure 1:**
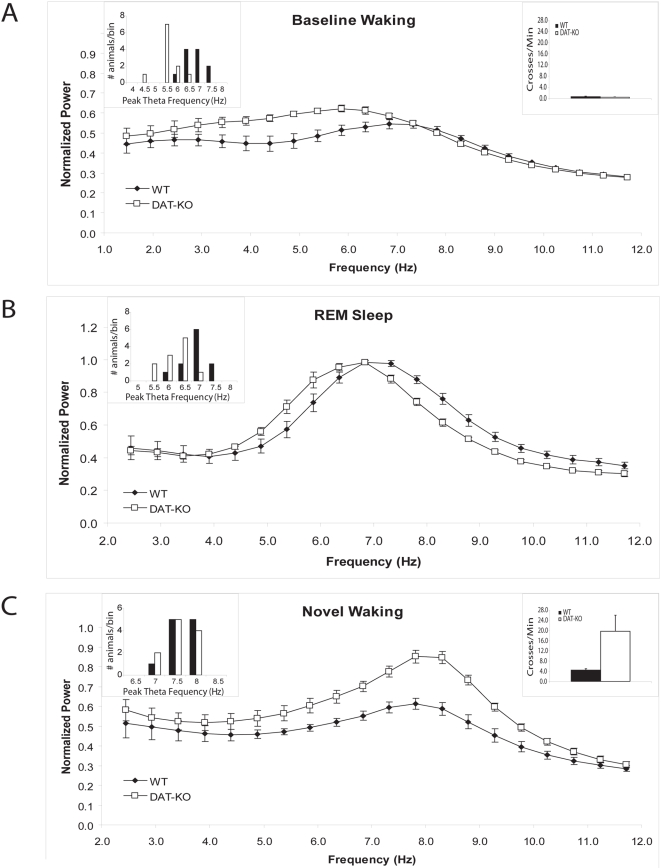
Altered HTOs in hyperdopaminergic mice. Mean hippocampal LFP power distributions were calculated in the 2–12 Hz range for each mouse across the behavioral periods of interest. The images depict normalized power spectral distributions averaged across animals within genotype. Two way ANOVA of HTO frequency found the main effects of genotype [F_1,49_ = 21.66, p<0.01] and condition [F_4,49_ = 82.82, p<0.01], as well as the genotype by condition interaction [F_4,49_ = 9.96, p<0.01] to be significant. Post-hoc tests revealed that DAT-KO mice display lower hippocampal theta oscillation (HTO) frequencies than WT mice during (A) baseline waking (p<0.01) and (B) REM sleep periods (p = 0.011), but not (C) waking periods immediately following novelty exposure (p = 0.35). Locomotor activity measured during the A) habituated and C) novelty exposed waking states are depicted in insets, and behavioral statistics are shown in Fig. 2F. One way ANOVA found significant effects when mice were exposed to a novel environment for both DAT-KO [F_4,24_ = 54.47, p<0.01] and WT mice [F_4,24_ = 31.19, p<0.001]. Newman-Keuls tests showed that peak HTO frequencies increased with novelty exposure for both genotypes (DAT-KO: N-K_20_ = 3.92, WT: N-K_20_ = 2.35). Error bars represent S.E.M for normalized theta power determined for each frequency across animals within a genotype; n = 11 for both genotypes. Two way ANOVA of total theta power found the main effects of genotype [F_1,39_ = 15.21, p<0.01] and condition [F_3,39_ = 4.55, p<0.01], as well as the genotype by wake-state interaction [F_3,39_ = 6.46, p<0.01] to be significant. *Post-hoc* tests revealed that total theta power was not different between DAT-KO and WT mice during the baseline waking period (p>0.1); however, it was significantly higher in DAT-KO mice than WT mice following novelty exposure (p<0.01). One way ANOVA's found significant effects of condition for DAT-KO [F_3,19_ = 8.22, p<0.01] but not WT mice [F_3,19_ = 2.05, p>0.05]. Newman-Keuls tests showed that novelty exposure increased total theta power in DAT-KO (N-K_10_ = 4.66).

**Figure 2:**
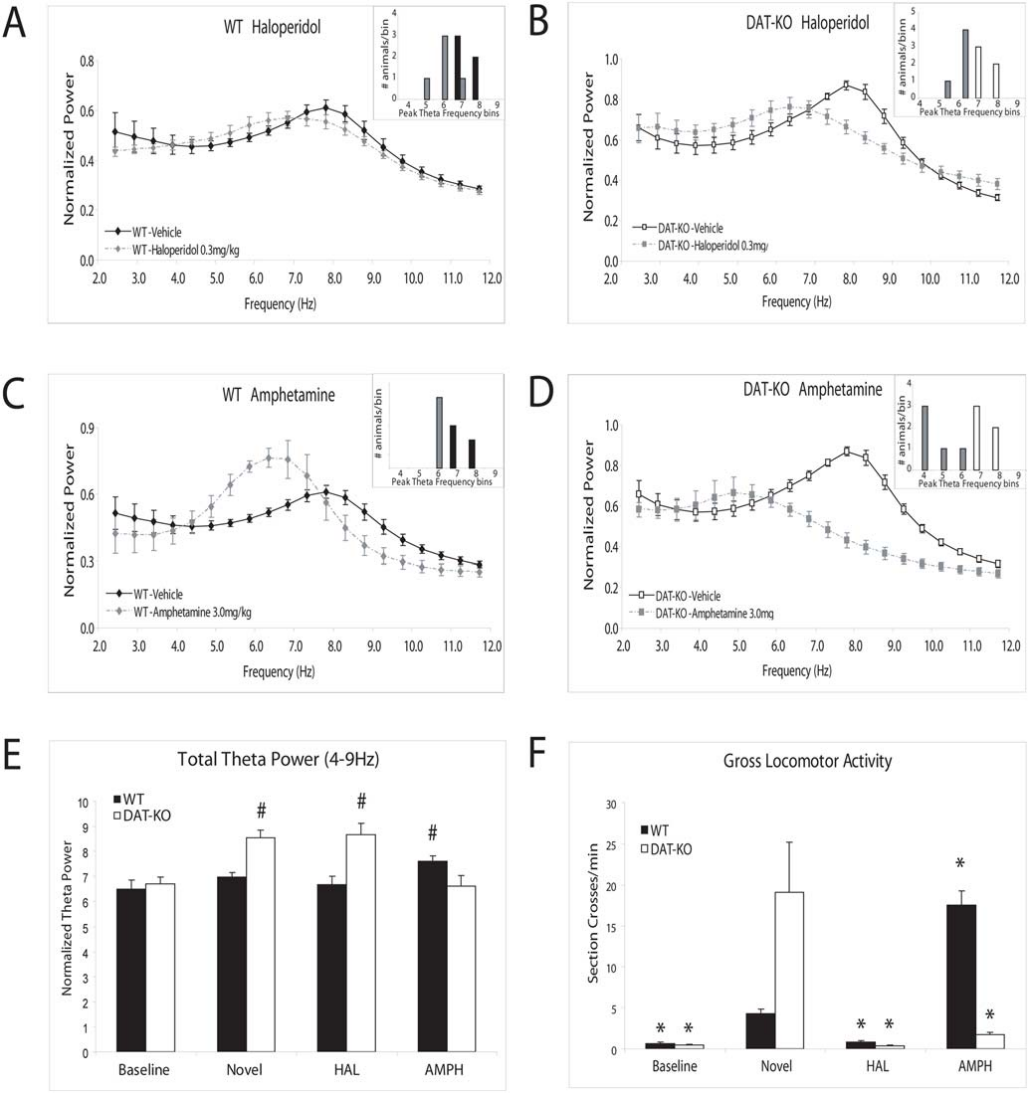
The effect of psychoactive agents on HTO frequencies in WT and DAT-KO mice. Hippocampal LFP power distributions were calculated in the 2–12 Hz range for each mouse across the drug treatment of interest. The images depict normalized power spectral distributions averaged across animals within genotype and drug treatment. Treatment with haloperidol 0.3 mg/kg (HAL) significantly attenuated novelty induced peak HTO frequencies increases in both (A) WT (N-K_20_ = 3.39) and (B) DAT-KO (N-K_20_ = 3.29) mice, and treatment with d-amphetamine 3.0 mg/kg (AMPH) attenuated novelty induced peak HTO frequency increases in both (C) WT (N-K_20_ = 3.06) and (D) DAT-KO (N-K_20_ = 4.42) mice. (E) Novelty and psychoactive drug effects on total theta power. Treatment with AMPH, but not HAL, potentiated novelty-induced theta power in WT mice (AMPH: N-K_20_ = 5.01, HAL: N-K_20_ = 5.01). Novelty-induced theta power was attenuated by treatment with AMPH, and unaffected by treatment with HAL in DAT-KO mice (AMPH: N-K_20_ = 5.66, and HAL: N-K_20_ = 4.66, respectively). (F) Novelty and psychoactive drug effects on locomotor activity. WT and DAT-KO mice displayed similar behavioral profiles during baseline periods (tstat_1,8_ = 0.96, p = 0.36). Novelty exposure induced behavioral hyperactivity in DAT-KO mice (tstat_1,8_ = 2.39, p<0.05), compared to novelty exposed WT mice. HAL attenuated locomotor activity in both genotypes. AMPH attenuated locomotor activity in DAT-KO mice, and potentiated locomotor activity in WT mice. Error bars represent S.E.M for recordings within a genotype. # = p<0.05; compared to animals within genotype during the baseline period. * = p<0.05; compared to animals within genotype during the novelty exposed; n = 5 for all groups.

DAT-KO mice display a period of marked behavioral hyperactivity lasting up to four hours after introduction to a novel environment [19], [24]. Thus, in order to examine the effects of persistent hyperdopaminergia on baseline HTOs, DAT-KO and WT mice were habituated to the recording chamber for eight hours and subsequently subjected to two-hour continuous electrophysiological and behavioral recordings. Despite WT and DAT-KO mice showing similar behavioral profiles, total delta power (normalized delta power: WT mice: 3.6±0.5, DAT-KO mice: 4.3±0.4; n = 5; p>0.05) and total theta power (see Fig. 1A and 2E), DAT-KO mice displayed lower peak HTO frequencies during the baseline waking period than WT mice (WT mice: 7.1±0.2 Hz, DAT-KO mice: 5.7±0.2 Hz; Fig. 1A,). In order to ensure that the lower HTO frequencies observed in DAT-KO mice were truly due to changes associated with persistent hyperdopaminergia, and not simply behavioral differences that were too small to measure with our scoring technique, we implored a second behavioral scoring method based on our video data and EMG recordings. Using this method for five animals in each group, we identified the minimal EMG activity associated with movement and limited our analysis to include only one second intervals where mice displayed EMG activity below the movement threshold. Importantly, DAT-KO mice continued to display lower peak HTO frequencies than WT mice when the analysis was restricted to periods when both groups were completely still (WT mice: 6.9±0.3 Hz, DAT-KO mice: 5.7±0.4 Hz; n = 5 per group). Thus, the lower HTO frequencies observed in behaviorally habituated DAT-KO mice were indeed due to changes associated with persistent hyperdopaminergia and not simply small differences in behavioral profiles. This result was particularly interesting because acute treatments with DA agonists has been shown to induce high frequency HTO's [9], [20], [27]. Next, we investigated if these changes in HTO's were present across all baseline behavioral states observed in DAT-KO mice by analyzing HTO spectral patters observed across DAT-KO and WT mice during REM sleep. Our results indicate that DAT-KO mice show lower peak HTO frequencies than WT mice during REM sleep (WT mice: 7.2±0.1 Hz, DAT-KO mice: 6.5±0.2 Hz; Fig. 1B). Importantly, similar results were also obtained when the frequency resolution was increased from 0.5 Hz to 0.25 Hz. This demonstrates that lower frequency HTO's are present across both baseline waking and sleeping periods in DAT-KO mice.

In order to determine if persistent hyperdopaminergia altered HTO activity during waking periods characterized by increased exploratory behavior, we conducted two-hour electrophysiological and behavioral recordings in DAT-KO and WT mice that were not habituated to the recording chamber. Interestingly, despite the marked behavioral hyperactivity displayed by the DAT-KO mice following exposure to the novel recording chamber, peak HTO frequencies were comparable to those observed in novelty-exposed WT mice (WT mice: 8.0±0.1 Hz, DAT-KO mice: 8.0±0.1 Hz, Fig. 1C). Peak HTO frequencies were, however, higher in novelty exposed WT and DAT-KO mice than those observed during the baseline waking period. Novelty exposure also increased theta power in DAT-KO mice, but not WT mice (Fig. 1C, see Fig. 2E). Importantly, exposure to novelty does not change dopaminergic tone in WT or DAT-KO mice [24]. Therefore, as WT and DAT-KO mice display similar peak HTO frequencies following exposure to novelty, while dopaminergic tone remains constant, our results suggest that persistent hyperdopaminergia does not decrease the peak frequency of HTO's in DAT-KO mice across all behavioral states.

Overall, our findings suggest that persistent hyperdopaminergia generates compensatory network changes which decrease the peak frequency of HTO's during baseline behavioral periods. Conversely, our results also raise the possibility that hyperdopaminergia lowers peak HTO frequencies across all behavioral states, and that the similar peak HTO frequencies observed in novelty exposed WT and DAT-KO mice is a result of the higher behavioral profiles exhibited by DAT-KO animals.

### Acute Dopamine D2 receptor transmission involved in novelty-induced increases in HTO frequencies

Next, we investigated the molecular mechanisms underlying the changes in HTO's observed in DAT-KO mice. DAT-KO mice and WT mice were treated with the D2 dopamine receptor antagonist haloperidol 0.3 mg/kg, i.p. (HAL), and two-hour electrophysiological and behavioral recordings were conducted. HAL attenuated behavioral hyperactivity (see Fig. 2F) and novelty-induced peak HTO frequency increases in both WT (vehicle: 8.0±0.1 Hz, HAL: 6.5±0.2 Hz; Fig. 2A) and DAT-KO mice (vehicle: 8.0±0.1 Hz, HAL: 6.3±0.1 Hz; Fig. 2B). Taken together, these findings demonstrate that acute dopaminergic transmission, via the D2 dopamine receptor, plays a critical role in the induction of high frequency HTO's in DAT-KO mice following novelty exposure. Interestingly, HAL did not reduce total theta power in DAT-KO mice (see Fig. 2E), suggesting that alterations in other neuromodulatory systems may mediate the novelty induced increases in theta power observed in these animals.

Next, we investigated the effect of HAL on the altered HTO's observed during baseline behavioral periods in DAT-KO mice. Systemic administration of pharmacological agents induced behavioral hyperactivity in habituated DAT-KO mice, thus we employed a modified experimental paradigm. Firstly, behavioral and electrophysiological recordings were extended to four hours in WT and DAT-KO mice treated with HAL. Secondly, analysis was limited to periods of REM sleep occurring within this four hour window. Because the four hour recording period was within the half-life of HAL in mice [28], and the effect of external behavioral influences on HTO's is relatively limited during REM sleep, this experimental paradigm allowed us quantify the effect of HAL on the altered HTO frequencies observed in habituated DAT-KO mice. Our results showed that DAT-KO mice treated with HAL displayed lower peak HTO frequencies during REM sleep that WT mice treated with HAL (WT mice: 7.5±0.3 Hz, DAT-KO mice: 6.6±0.1 Hz, p<0.02). These results indicate that HAL does not attenuate the altered HTO frequencies observed in DAT-KO mice during baseline behavioral periods.

### DA independent modulation of HTOs

Next, we conducted our recording protocol in WT mice treated with the psychostimulant d-amphetamine 3.0 mg/kg, i.p. (AMPH). Dopamine agonists have been shown to increase peak HTO frequencies [20]; thus, we predicted that treatment with AMPT would increase peak HTO frequencies in WT animals. Surprisingly, our results showed that WT mice treated with d-amphetamine displayed peak HTO frequencies that were significantly lower than those observed in WT mice treated with vehicle (WT: 6.7±0.1 Hz; tstat_1,8_ = 3.9, p<0.005, see Fig. 2C). We also found that AMPH increased total theta power in WT mice (see Fig. 2E). While AMPH causes a profound increase in dopamine release in WT animals at the dose used for this experiment (3.0 mg/kg), it has also been shown to increase serotonin levels [23]. Thus, we set out to investigate if the lower peak HTO frequencies observed in WT mice treated with AMPH resulted from acute hyperdopaminergia or secondary changes in other neuromodulatory systems.

Firstly, WT animals were treated with both HAL and AMPH, and electrophysiological data was collected during the 2-hour recording period. Our results showed that AMPH significantly lowered peak HTO frequencies in WT animals treated with HAL (WT/HAL and AMPH: 5.9±0.2 Hz; tstat_1,8_ = 2.9, p<0.02), indicting that HAL did not attenuate the peak HTO frequency lowering effect of AMPH. Secondly, we treated DAT-KO mice with d-amphetamine. DAT-KO mice lack a key target of AMPH, the DA transporter [21], and thus the drug exerts a calming effect via serotonergic mechanisms [23], [24]. In DAT-KO mice, AMPH significantly lowered peak HTO frequencies (vehicle: 8.0±0.1 Hz, AMPH: 5.3±0.4 Hz; Fig. 2D). Upon further analysis, we also found that DAT-KO mice treated with AMPH displayed peak HTO frequencies that were even lower than those observed in DAT-KO treated with HAL (N-K_10_ = 4.00). Incidentally, AMPH attenuated novelty-induced hyperactivity in DAT-KO mice to a lesser degree than HAL (HAL: 0.4±0.1, AMPH: 1.7±0.3, tstat_1,8_ = 3.9, p<0.005). Moreover, in contrast to HAL, treatment with AMPH also significantly attenuated novelty induced increases in total theta power in DAT-KO mice (see Fig. 2E). Finally, we treated WT animals with the serotonin selective reuptake inhibitor fluoxetine (FLU, 20 mg/kg i.p.). Our results showed that FLU significantly lowered the peak frequency of HTO's in WT mice (WT mice/Saline: 8.0±0.1 Hz, WT mice/FLU: 6.6±0.3 Hz, n = 5, p<0.05), suggesting that increases in serotonergic tone were sufficient to reduced HTO frequencies.

Taken together, these results suggest that the lower peak HTO frequencies observed in WT animals treated with AMPH are likely due to secondary changes in serotonergic neuromodulatory systems, and not acute hyperdopaminergia.

## Discussion

Our findings demonstrate that DAT-KO mice display decreased hippocampal theta oscillations frequencies during baseline waking periods and REM sleep. Incidentally, these findings are in contrast to studies that have shown that acute treatment with DA agonists generates increases in HTO frequencies [9], [20], [27]. Thus, our findings reveal for the first time that persistent hyperdopaminergia lowers baseline hippocampal theta oscillation frequencies.

HTO's are prominent LFP's generated by the brain, and occur in the 4–9 Hz frequency range. These oscillations are especially prominent during waking periods of increased attention and rapid-eye-movement (REM) sleep [1], [2]. HTO's control the timing of activity across neuronal populations in hippocampus, prefrontal cortex, and amygdala and coordinate gamma oscillatory activity [4], [5], [29]. Gamma oscillations are a major indicator of cortical processing and a critical determinant of long term potentiation and depression [30], [31]. Thus, even small changes in baseline HTO frequencies are likely to alter neural activity across large distributed brain networks, ultimately generating deficits in gross behavioral processes. Importantly, baseline HTO frequencies observed during quiet waking and REM sleep have been shown to be predictive of learning rates across rodents [32].

HTO's are also highly correlated with behaviors such as changes in posture or limb position, walking, head movements, and rearing [33]. We have previously shown that the enhanced theta power observed in DAT-KO mice following novelty exposure is not simply due to the increased locomotor activity observed in these animals [19]. While peak HTO frequencies increase in WT and DAT-KO mice following novelty-induced hyperactivity as well, the changes in baseline HTO's observed in DAT-KO mice are unlikely to be due to gross locomotor behavioral changes. Firstly, despite demonstrating similar behavioral profiles during the baseline waking period, DAT-KO mice display significantly lower the peak frequency of HTO's. Importantly, this difference persists even when our analysis is restricted to intervals when animals are completely still. Moreover, DAT-KO mice display lower peak HTO frequencies than WT mice during periods of REM sleep where both genotypes of mice are behaviorally inactive. WT and DAT-KO also display similar peak HTO frequencies following novelty exposure, though DAT-KO mice experience a significant increase in gross locomotor activity. Collectively, these findings suggest that the altered HTO frequencies observed in DAT-KO mice HTO's can be dissociated from changes in gross locomotor behavioral activity. Secondly, though treatment with AMPH induces gross locomotor hyperactivity in WT animals, it lowered peak theta oscillations frequencies. Moreover, treatment with d-amphetamine caused greater decreases in peak HTO frequencies in DAT-KO mice than treatment with haloperidol, though haloperidol attenuated behavioral hyperactivity to a greater degree. Taken together, these findings provide compelling evidence that persistent hyperdopaminergia generates changes in HTO's that are locomotor activity independent, resulting in a decrease in the peak frequency of HTO's during baseline periods.

DA is critically involved in regulating neural processes responsible for complex movements and emotions [18]. Consequently, altered central dopaminergic neurotransmission has been implicated in several neurological and psychiatric disorders such as schizophrenia, Parkinson's disease, and ADHD. While recent evidence demonstrates that transient over-expression of striatal D2 dopamine receptors during development results in deficits in working memory [34], and that increases in tonic dopaminergic stimulation generate global deficits in learning and memory [35], it is unclear how persistent changes in dopaminergic transmission alter neural activity in brain areas that mediate cognitive processes. Our results demonstrate that persistent hyperdopaminergia generates a baseline slowing of hippocampal neural networks. Additionally, we show that while administration of the classic antidopaminergic agent HAL attenuates behavioral hyperactivity in DAT-KO mice, it does not correct the reduced peak HTO frequencies observed during baseline behavioral periods.

Moreover, we show that treatment with AMPH attenuates novelty-induced behavioral hyperactivity, and reduces the peak frequency of HTO's and HTO power in persistently hyperdopaminergic mutants. DAT-KO mice lack a key target of AMPH, the DA transporter [21]; thus, it is likely that AMPH exerts a calming effect via serotonergic mechanisms [23], [24]. Recent evidence has shown that the stress cascade modulates central serotonergic signaling [36], and that disruption of serotonergic signaling mechanisms increase HTO power [16].

Taken together with our findings, this suggests that the baseline HTO alterations observed in DAT-KO mice may be mediated via changes in the serotonergic neuromodulatory system, and that the behavioral hyperactivity, and peak theta frequency and power increases observed in novelty exposed DAT-KO mice are likely mediated in-part by stress induced disruption of serotonergic mechanisms. Indeed, DAT-KO mice display profound alterations in serotonin homeostasis that may underlie the changes in baseline neuron-oscillatory properties presented here [25]. Thus, we propose that persistent hyperdopaminergia induces compensatory changes in serotonergic systems, in order to regulate information flow through circuits responsible for locomotor behavior [37], which result in slower processing rates in hippocampal neural networks. Overall, the present results show that DAT-KO mice display decreased HTO frequencies, and demonstrate the central role of persistent hyperdopaminergia in modulating baseline hippocampal neural oscillatory activity.
